# Time series analysis of age related cataract hospitalizations and phacoemulsification

**DOI:** 10.1186/1471-2415-6-2

**Published:** 2006-01-12

**Authors:** Alan M Leong, Eric J Crighton, Rahim Moineddin, Muhammad Mamdani, Ross EG Upshur

**Affiliations:** 1 Primary Care Research Unit, Sunnybrook and Women's College Health Sciences Centre, 2075 Bayview Ave, #E-349, Toronto, ON, M4N 3M5, Canada; 2 Department of Family and Community Medicine, University of Toronto, 256 McCaul Street, 2^nd ^Floor, Toronto, ON, M5T 2W5, Canada; 3 Department of Public Health Sciences, University of Toronto, McMurrich Building, 12 Queen's Park Crescent West, Toronto, ON, M5S 1A8, Canada; 4 Institute for Clinical Evaluative Sciences, 2075 Bayview Avenue, Toronto, ON, M4N 3M5, Canada; 5 Health Policy Management and Evaluation, University of Toronto, McMurrich Building, 2^nd ^Floor, 12 Queen's Park Crescent West, Toronto, ON, M5S 1A8, Canada; 6 Faculty of Pharmacy, University of Toronto, 19 Russell Street, Toronto, ON, M5S 2S2, Canada

## Abstract

**Background:**

Cataract surgery remains a commonly performed elective surgical procedure in the aging and the elderly. The purpose of this study was to utilize time series methodology to determine the temporal and seasonal variations and the strength of the seasonality in age-related (senile) cataract hospitalizations and phacoemulsification surgeries.

**Methods:**

A retrospective, cross-sectional time series analysis was used to assess the presence and strength of seasonal and temporal patterns of age-related cataract hospitalizations and phacoemulsification surgeries from April 1, 1991 to March 31, 2002. Hospital admission rates for senile cataract (*n *= 70,281) and phacoemulsification (*n *= 556,431) were examined to determine monthly rates of hospitalization per 100,000 population. Time series methodology was then applied to the monthly aggregates.

**Results:**

During the study period, age-related cataract hospitalizations in Ontario have declined from approximately 40 per 100,000 to only one per 100,000. Meanwhile, the use of phacoemulsification procedures has risen dramatically. The study found evidence of biannual peaks in both procedures during the spring and autumn months, and summer and winter troughs. Statistical analysis revealed significant overall seasonal patterns for both age-related cataract hospitalizations and phacoemulsifications (*p *< 0.01).

**Conclusion:**

This study illustrates the decline in age-related cataract hospitalizations in Ontario resulting from the shift to outpatient phacoemulsification surgery, and demonstrates the presence of biannual peaks (a characteristic indicative of seasonality), in hospitalization and phacoemulsification during the spring and autumn throughout the study period.

## Background

Cataract surgery represents one of the most common forms of elective surgery worldwide in persons over age 65, and one of the leading causes of hospital admissions in the province of Ontario [1,2]. In North America, cataracts afflict nearly one in six North Americans over the age of 40, approaching one in two by age 80. Cataracts are a major cause of blindness, although its etiology is still not fully understood [3,4]. Previous studies have demonstrated an empirical link between age-related cataracts and personal and environmental factors such as, UV radiation, heavy alcohol consumption, and smoking [3]. Predisposing conditions such as diabetes, glaucoma, myopia in early age, in addition to prior eye injuries, have also been linked to the development of cataracts [5]. Although non-life threatening, cataracts affect a person's ability to perform routine activities, lowering his or her overall quality of life. Cataract surgery in Ontario is paid for by the Ontario Health Insurance Program (OHIP) at no cost to the patient. In 2003–2004, the median wait time for cataract surgery in Ontario was found to be 15 weeks [6]. As a result, the efficacy of current treatments, coupled with the cost of health care, support the need for a thorough understanding of the patterns of cataract surgery in order to improve delivery of eye care services.

Studies examining the prevalence of cataracts have shown that the number of cataract operations have been growing since 1980 [7,8]. Rates of cataracts are linked to age, and with a burgeoning elderly population, the demand for surgery is likely to greatly increase [2,5]. Current literature on cataract hospitalizations is limited to analysis of single communities and has been performed only at annual levels. In order to address these limitations, this study employs a large population distributed over a vast geographical area, encompassing both urban and rural settings. Furthermore, this study examines monthly rates of cataract hospitalizations using population-based data for the province of Ontario over an 11-year period. Specifically, the objectives are: (1) to examine the overall trends in inpatient surgery versus outpatient surgery; (2) to determine if there are seasonal patterns of hospitalizations by gender; and (3) to assess the strength of these patterns.

## Methods

We conducted a retrospective, population-based study to assess temporal patterns in hospitalizations for age-related cataracts, and phacoemulsification and aspiration of cataract from April 1, 1991 to March 31, 2002. Approximately 14 million residents of Ontario eligible for universal health care coverage during this time were included for analysis. The Canadian Institute for Health Information (CIHI) Discharge Abstract Database was used to obtain information on hospitalizations for senile cataract (ICD-9 code: 366.1) and phacoemulsification (ICD-9 code: 13.41) as the principal diagnoses. This database records discharges from all Ontario acute care hospitals, documenting inpatient hospital stays with a scrambled patient identifier, date of admission and discharge, up to 16 diagnoses as coded by the International Classification of Diseases, Ninth Revision, Clinical Modification (ICD-9-CM), and up to 10 procedures. Researchers have found that diagnoses within this database are coded with a high degree of accuracy, with less than 1% of the basic information on patients missing [9-12].

All records with a principal discharge diagnosis of senile cataract (*n *= 70,281; male: 35%, female: 65%) and phacoemulsification (*n *= 556,431; male: 39%, female: 61%) were selected. The total number of discharges by gender were assessed for each month. Annual census data for each age group for residents of Ontario were provided by Statistics Canada. Monthly population estimates were derived through linear interpolation. Using this data, monthly hospitalization rates per 100,000 population were calculated and normalized for length of month. All transfers from one acute care hospital to another within this study group were excluded from the analysis.

Analysis of the data involved several statistical techniques in order to assess the statistical significance of seasonal patterns and the consistency and magnitude of seasonal effects. Spectral analysis was conducted to test for seasonality, detecting periodicity in time series, by plotting spectral density against period [13]. The data was detrended using moving averages of order 13 prior to conducting spectral analysis. Seasonality was tested using Fisher's κ (FK) and Bartlett Kolmogorov Smirnov (BKS) [14]. The autocorrelation function (ACF) was then used to measure the correlation between observations at different time lags [15]. A strong correlation between the observations at 12 time lags indicates a strong seasonality of the period 12. Finally, R-squared autoregression coefficients (*R*^2^_*Autoreg*_) were calculated. Autoregression uses the coefficient of determination (R^2^) of the autoregressive regression model fitted to the data, and can be used to measure the strength of seasonality within a set of serially correlated observations that occurs with time series data [16]. *R*^2^_*Autoreg *_is interpreted in the same way as the coefficient of determination in classical regression: 0–0.4 non-existent to weak seasonality, 0.4–0.7 moderate to strong seasonality, and 0.7–1.0 strong to perfect seasonality. All statistical analyses were performed using SAS 8.2 (SAS Institute Inc., Cary, NC, USA).

Ethical approval for this study was obtained from the Sunnybrook & Women's College Health Sciences Centre Research Ethics Board.

## Results

**Figure 1:**
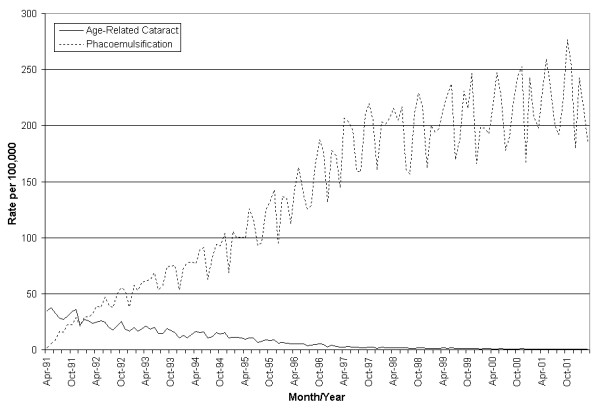
Time plot of age related cataract hospitalizations and phacoemulsification, aggregated by gender.

Figure 1 shows decreasing monthly hospitalization rates over the study period. The overall trend can be divided into two distinct periods. From 1991 through 1995, hospitalization rates dropped from 40 per 100,000 to 15 per 100,000. From 1995 to 2002, rates in autumn dropped from 15 per 100,000 to one per 100,000. From 1991 through 1995, rates routinely peak immediately after April and October with a relative drop in hospitalizations over the summer. In contrast, phacoemulsification procedures increased quickly from under 50 per 100,000 in 1991 to over 200 per 100,000 by the end of 2002. Gender analysis illustrates that hospitalization rates were higher among females than males throughout the study, and over time both genders fluctuate in near synchrony (data not shown).

**Figure 2:**
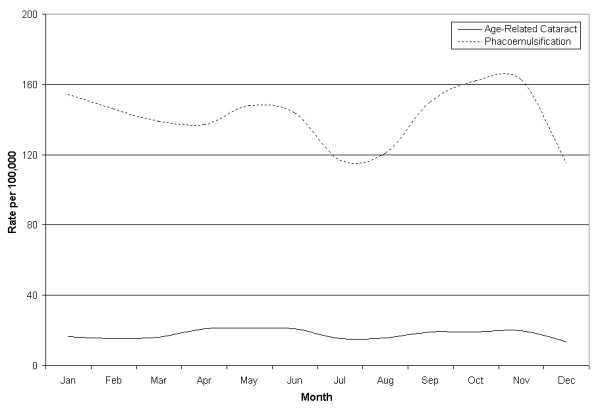
Age related cataract hospitalizations and phacoemulsification, aggregated by month.

The seasonal distributions of age-related cataract hospitalizations and phaocemulsification surgeries for both genders are illustrated in Figure 2. Biannual peaks are present in both cataract hospitalizations and phacoemulsifications. Peak phases are more prominent during the spring and less prominent in the autumn throughout the study. Spring hospitalization phases (21 per 100,000) were slightly higher than autumn peaks (19 per 100,000). Peak phacoemulsification phases differed greatly between the spring (140 per 100,000) and autumn (158 per 100,000). However, both hospitalizations and phacoemulsifications have troughs between peak phases.

Results of the time series analysis are seen in Table 1. Fisher's κ demonstrates significant seasonality (*p *< 0.01) in both genders overall for hospitalizations and phacoemulsifications, as well as by gender. Similarly, BKS showed significant seasonality (*p *< 0.01). The R^2^_*Autoreg *_value for both genders combined was 0.64 for hospitalizations, suggesting moderately strong seasonality, and 0.78 for phacoemulsification, indicating strong seasonality.

**Table 1:** Seasonality of age-related cataract hospitalizations and phacoemulsification by gender.

	Fisher's κ*			**BKS^†^**			**R^2 ^Autoregression Log Transformed**		
	**Male**	**Female**	**Both**	**Male**	**Female**	**Both**	**Male**	**Female**	**Both**
Age-related cataract hospitalization	18.44^‡^	18.68^‡^	24.11^‡^	0.22^‡^	0.25^‡^	0.27^‡^	0.43	0.52	0.64
Phacoemulsification	16.44^‡^	16.67^‡^	16.64^‡^	0.26^‡^	0.27^‡^	0.27^‡^	0.74	0.79	0.78

* Tests the null hypothesis that the series is Gaussian white noise against the alternative hypothesis that the series contains an added deterministic periodic component of unspecified frequency.

^† ^Tests the null hypothesis that the series is white noise.

^‡ ^*p *< 0.01.

FK, Fisher's κ; BKS, Bartlett's Kolmogorov-Smirnov

## Discussion

This study revealed several important findings. Age-related cataract hospitalizations were found to peak in the spring and autumn, and drop during the summer and winter. Further analysis revealed that overall seasonal patterns were statistically significant for both hospitalizations and phacoemulsifications. The most striking finding is the downward trend in age-related cataract hospitalizations concurrent with a significant rise in phacoemulsification over the study, a finding consistent for both genders.

Similar patterns of hospitalization are seen in otolaryngological day surgery, where, for example, nasal polyp extractions (a common elective procedure) were found to peak in April-June, drop significantly during midsummer, and peak again in September-October [17]. The troughs in hospitalizations coincide with the months when physicians and surgeons request vacation time, and are therefore unlikely to schedule elective surgical procedures (i.e. July, August and December). This is seen in Scotland, where the lowest elective surgical activity occurs during summer months (July through September) [18].

Over the course of this study, hospitalization rates for age-related cataracts decreased from approximately 50–55 per 100,000 to roughly one to two per 100,000, while phacoemulsification grew more than 100-fold. Previous studies set precedence for such an occurrence [7,19-22]. As an elective surgical procedure, the reduced surgical times, in addition to other benefits, such as lower costs for the procedure and improved surgical outcome, may serve to further motivate patients to undergo surgery [7]. A rise in phacoemulsification procedures is the likely explanation for the downward trend in hospitalizations (Figure 1).

A few limitations of our study should be noted. The current study is descriptive in nature and thereby does not fully address potential explanatory factors for the hosptialization patterns. In addition, we are unable to measure temporal trends or seasonal variation in the use of other health services such as emergency department or physician visits. We have counted each procedure in the numerator, although one person could have two procedures. However, it is exceedingly unlikely for an individual to have two cataract procedures at the same time, or within the same calendar month so this does not bias the measures. The strengths of this study lie in its longitudinal base and large population size coupled with the use of a comprehensive time series analysis approach applied to gender. These results improve our understanding of gender differences as well as overall trends in age-related cataract hospitalizations in Ontario.

## Conclusion

This study serves to illustrate the decline of age-related cataract hospitalizations and rise of phacoemulsification surgery, plus the existence of a significant seasonal pattern in age-related cataract hospitalizations. The evidence collected supports the notion that elective surgical procedures are not seasonally driven by disease prevalence, but in fact driven by extrinsic factors, such as physician vacation periods, hospital administrations which regulate surgical schedules, and patients evaluating treatment options. Further time series analysis of patterns of elective procedures can illustrate the distribution of elective surgeries throughout a calendar year. Such data can be used by health care providers for future cost analyses and allocation of services effectively based on seasonal patterns of system utilization.

## Competing interests

The authors declare that they have no competing interests.

## Authors' contributions

AML participated in the design of the study, analysis and interpretation of the data, and prepared the first draft of the manuscript. EJC participated in the analysis and interpretation of the data. RM provided statistical expertise in the analysis and interpretation of the data. MM participated in the analysis and interpretation of the data. REGU initiated and designed the study, and will act as guarantor. All authors contributed to the editing and revising of the manuscript. All authors have read and approved the final version.

## Pre-publication history

The pre-publication history for this paper can be accessed here:


